# *PpSARK* Regulates Moss Senescence and Salt Tolerance through ABA Related Pathway

**DOI:** 10.3390/ijms19092609

**Published:** 2018-09-03

**Authors:** Ping Li, Hong Yang, Gaojing Liu, Wenzhang Ma, Chuanhong Li, Heqiang Huo, Jianfang He, Li Liu

**Affiliations:** 1 Key Laboratory of Economic Plants and Biotechnology, Germplasm Bank of Wild Species, Kunming Institute of Botany, Chinese Academy of Sciences, Yunnan Key Laboratory for Wild Plant Resources, Kunming 650201, China; liping@mail.kib.ac.cn (P.L.); yanghong@mail.kib.ac.cn (H.Y.); liugaojing@mail.kib.ac.cn (G.L.); mawenzhang@mail.kib.ac.cn (W.M.); hejianfang@mail.kib.ac.cn (J.H.); 2 University of Chinese Academy of Sciences, Beijing 100049, China; 3 National Key Laboratory of Crop Genetic Improvement and National Centre of Plant Gene Research, Huazhong Agricultural University, Wuhan 430070, China; lchh5@webmail.hzau.edu.cn; 4 Mid-Florida Research and Education Center, Department of Environmental Horticulture, University of Florida, Apopka, FL 32703, USA; hhuo@ufl.edu

**Keywords:** senescence, salt stress response, ABA, SARK, moss (*Physcomitrella patens*)

## Abstract

Senescence-associated receptor-like kinase (SARK) family members in *Arabidopsis*, soybean, and rice are known to be positive regulators of leaf senescence. In the meantime, SARKs are extensively involved in stress response. However, their function and underlying molecular mechanism in stress responses in moss are not well known. Here, we investigated functional roles of *SARK* isolated from *Physcomitrella patens* (*PpSARK*) in salt stress response and senescence. *PpSARK* transcripts significantly accumulated under NaCl and abscisic acid (ABA) treatments, with higher expression in the moss gametophyte stage. Insertional gain-of-function mutants of *PpSARK* (*PpSARKg*) were more tolerant to salt stress and ABA than wild type (WT), whereas senescence of mutants was delayed during the protonema stage. Expression of stress-responsive genes in the ABA related pathway, such as *PpABI3*, *PpABI5*, *PpPP2C*, and *PpLEA* were significantly higher in *PpSARKg* and WT under salt stress conditions, suggesting that *PpSARK* might positively regulate salt tolerance via an ABA-related pathway. Endogenous ABA contents also increased 3-fold under salt stress conditions. These results indicate that *PpSARK* functions as a positive regulator in salt stress responses, while possibly functioning as a negative regulator in senescence in moss.

## 1. Introduction

Leaf senescence is the final stage of leaf developmental processes that are influenced by both internal genetic factors and external environmental cues such as temperature [1]. Phytohormones abscisic acid (ABA), jasmonic acid (JA), and salicylic acid (SA) could positively regulate leaf senescence. Overexpression of ABA receptor *PYL9* driven by the *RD29A* promoter resulted in better drought tolerance and accelerated leaf senescence. The *PYL9* promoter, ABA, induced leaf senescence and drought tolerance via inhibiting PP2Cs and activating SnRK2s [2]. Thousands of senescence-associated genes were revealed by extensive genomic studies related to senescence inducers [3,4]. Liu and Li developed a leaf senescence database (LSD; available online: http://www.eplantsenescence.org/) that contains a total of 5357 senescence-associated genes (SAGs) from 44 species based on experimental evidence from the literature [5,6]. Other than TFs such as members of the WRKY, NAC, MYB, bHLH, and bZIP families, kinase-domain proteins can be found in the LSD [7,8]. Senescence-associated receptor-like kinases (SARKs) are widely reported to be involved in abiotic and biotic stress resistance [9]. Salt stress causes severe crop failure. The *Oryza sativa* salt tolerance activation 2-dominant (*OsSta2-D*) gene and some microRNAs were found with functions for enhancing plant salt tolerance in rice and maize [10,11]. Salt stress may damage photosynthetic systems and accelerate leaf senescence via accumulating toxicity in the cell cytosol. Crosslinks between ROS and ABA (abscisic acid)-dependent signaling, together with ion homeostasis and the sumoylation pathways in plant salt and drought tolerance were established [12,13,14]. RNA sequencing (RNA-seq) and small RNA sequencing (sRNA-seq) data of citrus roots also confirmed that signal transduction, hormone-mediated signaling pathways, ROS metabolic processes, and transcription factors were involved in dehydration and/or salt treatment [15]. Most of the progress in understanding the crosstalk of senescence and stress resistance comes from the model dicot plant *Arabidopsis thaliana*, while limited information is known about moss mechanisms [16].

Senescence-associated receptor-like kinases (SARKs) were first named in bean (*Phaseolus vulgaris*) and they were characterized from a leaf-senescence complementary DNA (cDNA) library [17]. These membrane-localized serine/threonine protein kinases with a leucine-rich repeat in the extracellular region are quite conserved among plants, animals, and microorganisms. They play a role in various cellular processes via catalyzing protein phosphorylation to regulate stress response genes. *Arabidopsis thaliana* *AtSARK* (At4g30520) and *Glycine max GmSARK* (AY687391) were previously studied for their regulation of leaf senescence through the synergistic actions of auxin and ethylene [18]. The cytoplasmic domain of AtSARK can be dephosphorylated by a PP2C-type protein phosphatase, senescence-suppressed protein phosphatase (SSPP) [19]. The overexpression of *IPT* (isopentenyltransferase) gene under the promoter of bean *SARK* (P_SARK_-IPT) increased plant drought tolerance and delayed drought-induced senescence [9,20,21].

Mosses, which were early land plants, share many conserved pathways among green plants. However, a lot of divergence exists in conserved families [22,23]. Here, we found an interesting role of *PpSARK*, which shared 70% homology with bean SARK. Insertional gain-of-function mutants of *PpSARK* were more tolerant to salt stress and ABA than wild type (WT), whereas senescence of mutants was delayed during the protonema stage. *PpSARK* possibly functions as a positive regulator in salt stress responses, while functioning as a negative regulator in senescence. It displayed an unknown, but quite different, pathway for regulating senescence and salt resistance compared to other higher green plants.

## 2. Results

### 2.1. PpSARK Is a Development-Associated Gene Induced by ABA and NaCl

**Figure 1:**
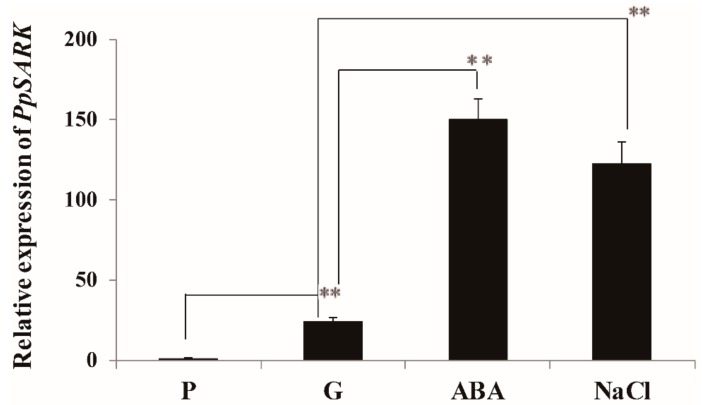
*SARK* isolated from *Physcomitrella patens* (*PpSARK*) is a development-associated gene induced by abscisic acid (ABA) and NaCl. P—protonema tissue at five days old; G—gametophyte at one month old. The gametophyte was treated with ABA and NaCl. Data are represented as means ± SD, *n* = 3. The *t*-test was used and an asterisk indicates that the value of treatment is different from the control; ** *p* < 0.01.

We previously carried out sequence alignment and material consultation in the database (available online: https://phytozome.jgi.doe.gov/pz/portal.html) and found one homolog of bean SARK (Pp3c22_12040) in *Physcomitrella patens*. To explore the function of *PpSARK* (Pp3c22_12040), we firstly investigated the expression pattern of *PpSARK* in wild-type moss and identified that *PpSARK* was induced by gametophore development (which is the second stage of moss development, prior to protonema), ABA, and salt stress treatment (Figure 1). The expression of *PpSARK* increased 4-fold under ABA, and 3-fold under salt treatment in gametophores. This result suggests that *PpSARK* is a senescence-associated gene, which mainly functions in the gametophores of moss and may be induced by ABA and NaCl. It is possible that *PpSARK* positively regulates moss salt resistance through the crosstalk of an ABA-related pathway.

### 2.2. PpSARK Regulates Moss Senescence and Salt Tolerance

**Figure 2:**
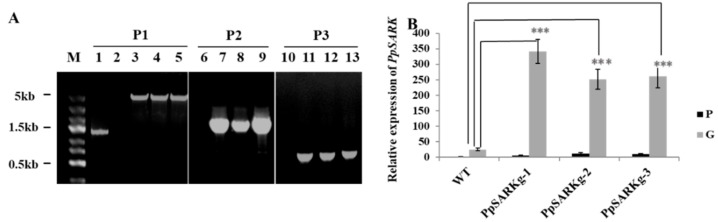
*PpSARKg* are gain-of-function mutants. (**A**) The genomic amplification of *PpSARKg* plants. Lanes 1, 6, and 10, products of wild-type (WT) genomic DNA samples; Lane 2, negative PCR control; Lanes 3, 7, and 11, products of *PpSARKg-1* genomic DNA samples; Lanes 4, 8, and 12, products of *PpSARKg-2* genomic DNA samples; Lanes 5, 9, and 13, products of *PpSARKg-3* genomic DNA samples. DNA fragments (F) were amplified by three primer sets: P1—F1/R2; P2—F1/R1; P3—F2/R2. Primer sequences used for each PCR are indicated in Table S1. (**B**) Quantitative analysis of the expression levels of *PpSARK* increased in *PpSARKg* plants. P—protonema tissue at five days old; G—gametophyte at one month old. Data are represented as means ± SD, *n* = 3. The *t*-test was used and an asterisk indicates that the value of treatment is different from the control (WT); *** *p* < 0.001.

To further understand the function of *PpSARK*, we next examined whether the *PpSARK* gene has any potential roles in the development of salt stress tolerance and plant senescence. We first attempted to generate knockout transgenic plants through homologous recombination (HR) by transforming pTN182-*PpSARK* into moss protoplasts (Figure S1A,B). Three knockout mutants for *PpSARK* were obtained by screening 50 moss transgenic lines using the PCR strategy illustrated in Figure S1B. We initially expected that genomic *PpSARK* would be replaced via HR of the KO construct (Figure S1A,B). As shown in Figure 2, the transforming DNA fragment underwent targeted insertion and integrated at one end of the targeted locus via HR (Figure 2A and Figure S1C). Surprisingly, the expression of *PpSARK* increased more than 5-fold in the protonema and gametophores of the three lines, and remarkably, it even accumulated more than 10-fold in the gametophores of the three lines than seen in the wild type (Figure 2B). These results indicated that the three transgenic lines were gain-of-function mutants (*PpSARKg*).

**Figure 3:**
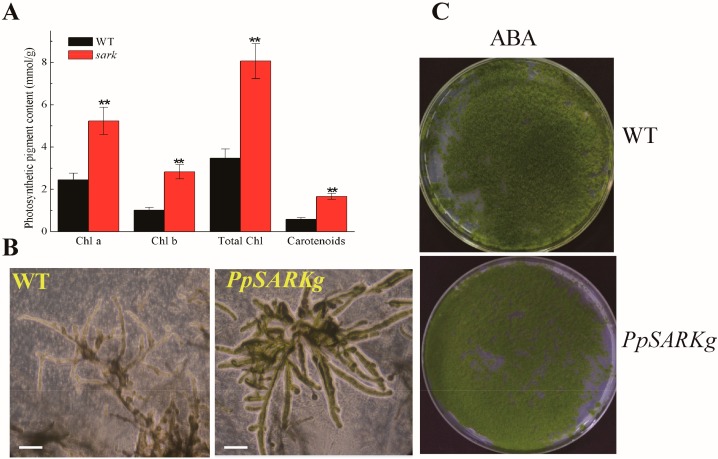
*PpSARK* regulated senescence in protonema tissues. (**A**) Pigments increased in the protonema of *PpSARKg*. Chl a—chlorophyll a; Chl b—chlorophyll b; Total Chl—Total chlorophyll. (**B**) Protonema of *PpSARKg* delayed senescence. (**C**) *PpSARKg* were not sensitive to extra ABA treatment. Data are represented as means ± SD, *n* = 3. The *t*-test was used and an asterisk indicates that the value of treatment is different from the control (WT); ** *p* < 0.01. The scale bar in (**B**) = 100 nm.

In addition, we tested the function of *PpSARK* in the protonema of *PpSARKg* and wild type, on the basis of photosynthetic pigment content and microexamination. We found that *PpSARKg* plants displayed delayed senescence symptoms in 10-day-old protonema (Figure 3A,B). Contents of photosynthetic pigments such as chlorophyll a and b, as well as total chlorophyll and carotenoids, were significantly higher in *PpSARKg* mutants (Figure 3A). The *PpSARKg* line grew stronger and had more green branches than seen in the wild type in 10-day-old protonema (Figure 3B). ABA was reported to be one of the hormones that can induce plant senescence [3]; hence, we treated five-day-old *PpSARKg* and wild-type protonema with extrogenous 100 µM ABA for three days to check the effect on the induction of senescence. The wild type was sensitive to ABA and was seen to yellow after ABA treatment, while the *PpSARKg* line was green (Figure 3C). The result showed that wild-type plants showed obviously accelerated senescence, while *PpSARKg* plants were not sensitive to ABA.

**Figure 4:**
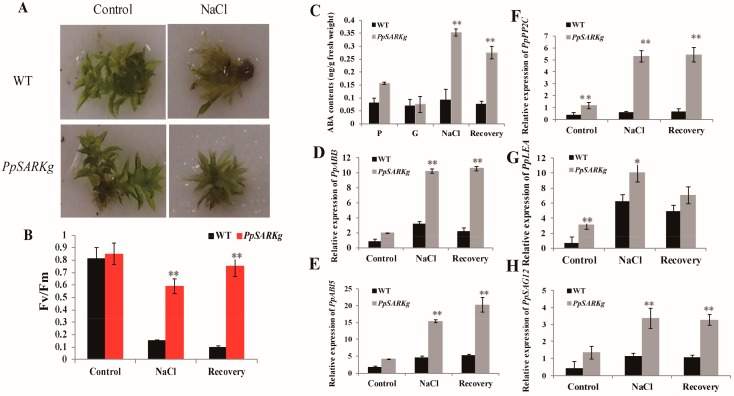
*PpSARK* regulates salt resistance and senescence via an ABA-related pathway. (**A**) *PpSARKg* plants were tolerant to salt stress. (**B**) Photosystem efficiency maintained during the salt treatment in gametophores of *PpSARKg* plants. Data are represented as means ± SD, *n* = 3. The *t*-test was used and an asterisk indicates that the value of treatment is different from the control (WT); ** *p* < 0.01. (**C**) Endogenous ABA contents of *PpSARKg* plants; three biological replicates with at least three technical repeats were done. P—protonema tissue at five days old; G—gametophyte at one month old. (**D**–**H**) The expression of marker genes in the ABA signaling pathway and senescence were induced in salt stress treatment. Data are represented as means ± SD, *n* = 3. The *t*-test was used and an asterisk indicates that the value of treatment is different from the control (WT); * *p* < 0.05; ** *p* < 0.01.

Given the distinct responses to ABA observed between *PpSARKg* and wild type, we wondered if other stresses, such as salinity, may also induce distinct responses between the two genotypes, since salinity can greatly induce ABA accumulation. We then investigated the response of *PpSARKg* plants to salt stress. One-month-old gametophores of *PpSARKg* and wild type were treated with 500 mM NaCl for three days. Under salinity treatment, *PpSARKg* grew a little stronger than wild-type plants, a difference which was not obvious. However, tissue bleaching and the loss of the photosynthetic activity and pigments were observed in wild-type plants after being transferred to the recovery medium (Figure 4A,B). By contrast, *PpSARKg* plants gradually turned white in the first five days after being transferred to the recovery medium, before turning green in the subsequent week. Its photosynthetic activity retained significantly high levels during NaCl treatment, and it totally recovered after been transferred to the recovery medium (Figure 4B). These results indicate *PpSARKg* plants are tolerant to salt stress.

### 2.3. PpSARK Regulates Salt Tolerance and Senescence via an ABA-Related Pathway

To further explore the underlying regulatory molecular mechanism of *PpSARK*, we performed qRT-PCR analysis of the ABA pathway or senescence marker genes and measured endogenous ABA contents during senescence and salt stress treatment. Both in the wild-type and *PpSARKg* plants, the expressions of *PpABI3*, *PpABI5*, and *PpPP2C* were rapidly induced by salt stress and recovery. However, more ABA-signaling-related messenger RNAs (mRNAs; *PpABI3*, *PpABI5*, and *PpPP2C*) were significantly accumulated in *PpSARKg* plants than in wild type. The relative expression levels of *PpABI3*, *PpABI5*, and *PpPP2C* in *PpSARKg* plants increased at least 2-fold during salinity treatment and recovery than in wild type (Figure 4D–F). The downstream functional gene (*PpLEA*) was also accumulated in *PpSARKg* plants (Figure 4G). These results indicated that the plants obtained higher salt stress tolerance related to ABA signaling. Endogenous ABA contents accumulated 2-fold in protonema cells, while they accumulated 3- to 4-fold during salt stress and recovery. However, endogenous ABA showed more or less the same levels in gametophore cells of wild-type and *PpSARKg* plants (Figure 4), while the expression of *PpSAG12* significantly increased 3- to 5-fold during salt stress treatment and recovery. The presence of enough ABA in protonema potentially resulted in the *PpSARKg* plants not being sensitive to ABA treatment. These results suggest *PpSARK* took part in the ABA pathway and possibly regulates salt tolerance and senescence via an ABA-related pathway.

## 3. Discussion

Moss plants harbor conserved gene families among land plants [24]. *SARK* is a large gene family and underwent multiple occurrences of gene duplication during evolution (Figure S2). In higher green plants, *SARK* was reported to positively regulate leaf senescence through hormone crosstalk, whereas research on salt-response-related functions of *SARK* is limited [18,19]. Inducible overexpression of *AtSARK* or *GmASRK* led to precocious leaf senescence, breakdown of chloroplast structure, and abnormal flower morphology [18]. An interesting work on *SARK* came from the promoter of bean *SARK* (P_SARK_), which was induced by leaf senescence and drought stress treatment [17,21]. Expressing IPT under the control of P_SARK_ (P_SARK_-IPT) could significantly enhance rice, tobacco, and peanut drought tolerance, and delayed drought-induced senescence via cytokinin-dependent photorespiration elevation and photosynthesis protection [9,20,21,25].

Here, we studied the function of the moss homolog of bean *SARK*, *PpSARK*. RNA transcripts of *PpSARK* were induced by gametophore development, as well as ABA and salt stress treatment (Figure 1). Different from functions in other plants, the salt resistance of *PpSARKg* plants was significantly enhanced. The protonema cells of gain-of-function mutants, *PpSARKg*, were not sensitive to ABA treatment, while the gametophore cells enhanced tolerance to salt stress treatment (Figure 4). Nevertheless, the protonema cells of *PpSARKg* displayed delayed senescence symptoms in one-week-old protonema cells (Figure 3A), probably due to the development stage transition in moss (*Physcomitrella patens*) [26]. As previously reported, the first protonema cells produced by spores are called primary chloronema, shortly followed by caulonema cell formation. Chloronema play primarily assimilatory roles and grow slowly, while caulonema absorb nutrients, grow fast, and initiate gametophore shoots. These developmental stages are highly regulated by auxin homeostasis, perception, and signaling [26]. The protonema tissues are quite different from leaves of other higher plants; we predicted *PpSARK* could be a negative regulator in moss protonema senescence, because of the important role of the development of protonema from single cells in moss. In our study, endogenous ABA contents increased in *PpSARKg* plants, demonstrating that *PpSARK* is probably involved in ABA signaling. Further analyses on *PpSARK*-mediated salt response revealed the involvement of an ABA-related pathway in the regulation of this process (Figure 4). A possible role for *PpSARK* in the regulation of senescence and salt resistance should be considered.

The moss *P. patens* is well known for its high frequency of gene targeting via HR [27]. Yet, targeted insertion with one end or untargeted integration at non-homologous sites occur at a certain frequency in DNA transformation [28]. In our study, transformed DNA underwent integration at 3’ end of the targeted locus via HR. We predict this event may be due to the short HR fragments used for transformation. Recent progress on moss genome editing technologies with the CRISPR-Cas9 system provides a powerful tool for studying the function of gene families with numerous members [29,30]. The differentiation of gene function from its origin is very interesting and important for deeply understanding the colonization of land by plants.

## 4. Materials and Methods

### 4.1. Moss Growth and Transformation

*Physcomitrella patens* (Grandson WT62) wild type plants [31] were grown on BCD medium supplemented with 5 mM ammonium tartrate and 1 mM CaCl_2_, and were overlaid with cellophane at 22 °C under continuous light (60 to 80 μM photons m^−2^·s^−1^) for one week, before being transferred on the growth matrix block for two weeks to get gametophytes. Polyethylene-glycol-mediated transformation of protoplasts was performed to generate the transgenic plants, as described by Shi and Theg [31].

### 4.2. Plasmid Construction and Genotyping

Moss *PpSARK* genomic DNA was used to amplify the upstream (the first 506 bp from ATG) and downstream (the last 513 bp from TAA) fragments. The upstream fragment was cloned to the SalI/EcoRI sites of the vector pTN182, and the downstream fragment was cloned to the XbaI/BamHI sites. The primers used for the upstream fragment were *PpSARK* uF and *PpSARK* uR, while those used for the downstream fragment were *PpSARK* dF and *PpSARK* dR. For genotyping, primers located in upstream and downstream sites were used as indicated in Figure S1 and Table S1.

### 4.3. Stress Treatments

To test the expression of *PpSARK* during stress treatment, wild-type, one-month-old gametophores were transferred onto 500 mM NaCl-saturated filter paper and 100 µM ABA, which were maintained for three days, followed by recovery on BCD medium for two weeks. For the salt stress treatment, one-month-old gametophores were transferred onto 500 mM NaCl-saturated filter paper and maintained for three days, followed by recovery on BCD medium for two weeks. For ABA stress treatment, 100 µM ABA was supplied in BCD medium for the treatment of five-day-old protonema tissues for three days.

### 4.4. Measurement of Chlorophyll Fluorescence (Fv/Fm) and Chlorophyll Content

Chlorophyll florescence of leafy gametophores was monitored using an IMAGING-PAM chlorophyll fluorometer and the Imaging Win software application (Walz, Effeltrich, Germany), was measured during salt treatment and recovery [32]. Pigments were isolated from the leafy gametophores with non volatile *N*,*N*-dimethylformamide (DMF) and measured with a Tecan Infinite M200 Pro (TECAN, Grödig, Austria) [33].

### 4.5. Measurement of Endogenous ABA

Phytohormones ABA was extracted from 0.1–0.3 g of frozen protonema or gametophores as described by Cai et al. [34]. After phytohormone extraction, the phytohormone content was measured using HPLC (Shimadzu, Kyoto, Japan) [35].

### 4.6. RNA Isolation, cDNA Synthesis, and Quantitative RT-PCR

Total RNA from moss tissues was isolated using TRIzol (Invitrogen, Carlsbad, CA, USA) following the manufacturer’s instructions. One microgram of RNA was treated with DNaseI and reverse-transcribed with oligo (dT) using a PrimeScript™ RT reagent Kit with gDNA Eraser (Takara, Dalian, China). The relative expression levels of individual genes were measured with gene-specific primers using quantitative real-time PCR (qRT-PCR) analysis, which was carried out in a 20-µL reaction mix with 1 µL of diluted cDNA template and SYBR Premix Ex TaqII (Takara, Dalian, China) with Bio-Rad CFX96. The adenine phosphoribosyl transferase gene (*PpAPT*) served as the internal control. The relative expression levels of target genes were calculated using the Bio Rad CFX96 system on the control of the expression of *PpAPT.*

## Supplementary Materials

The Supplementary Materials are available online at http://www.mdpi.com/1422-0067/19/9/2609/s1.

## Accession Numbers

Gene data used in this article can be found in Phytozome v12.1 (available online: https://phytozome.jgi.doe.gov/pz/portal.html) under the following accession numbers: *PpSARK*, Pp3c22_12040; *PpABI3*, Pp3c2_3370; *PpABI5*, Pp3c20_7230; *PpPP2C*, Pp3c7_5390; *PpLEA*, Pp3c19_7890; *PpSAG12*, Pp3c21_12340; and *PpAPT*, Pp3c8_16590.

## Abbreviations

SARK	Senescence-associated receptor-like kinase
