# Supplementary material for: PpSARK Regulates Moss Senescence and Salt Tolerance through ABA Related Pathway
Source: Int J Mol Sci. 2018 Sep 3;19(9):2609. doi: 10.3390/ijms19092609 (PMC6163601; doi:10.3390/ijms19092609)
Supplement: Supplementary file 1 [file ijms-19-02609-s001.pdf]

Supplementary Materials

PpSARK Regulates Moss Senescence and Salt Tolerance through ABA-Related Pathway

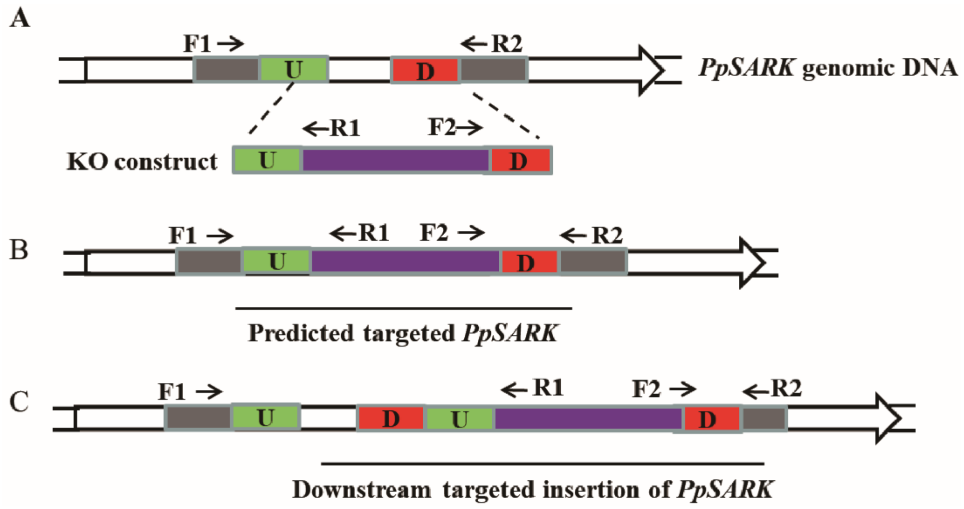

Figure S1. Models for the targeted insertion of *PpSARK*. (**A**) Schematic representation of a *PpSARK* locus with a targeting construct (knockout (KO) construct). (**B**) Predicted knockout event of integration via homologous recombination (HR) in both arms of the vector. (**C**) The transforming fragments integrated via HR in the downstream arm and the upstream arm invade the breakpoint to integrate via non-homologous end joining (NHEJ). Positions of primers used for PCR are shown. The lengths of DNA fragments: U (upstream arm)—506bp; D (downstream arm)—513bp; predicted knockout DNA fragment—772bp; NptII backbone—2.1kb; P1 (F1/R2) in (**B**)—3.1kb; P1 in (**C**)—4.9kb; P1 in WT—1.8kb; P2 (F1/R1) in (**B**)—556bp; P2 in (**C**)—2.3kb.

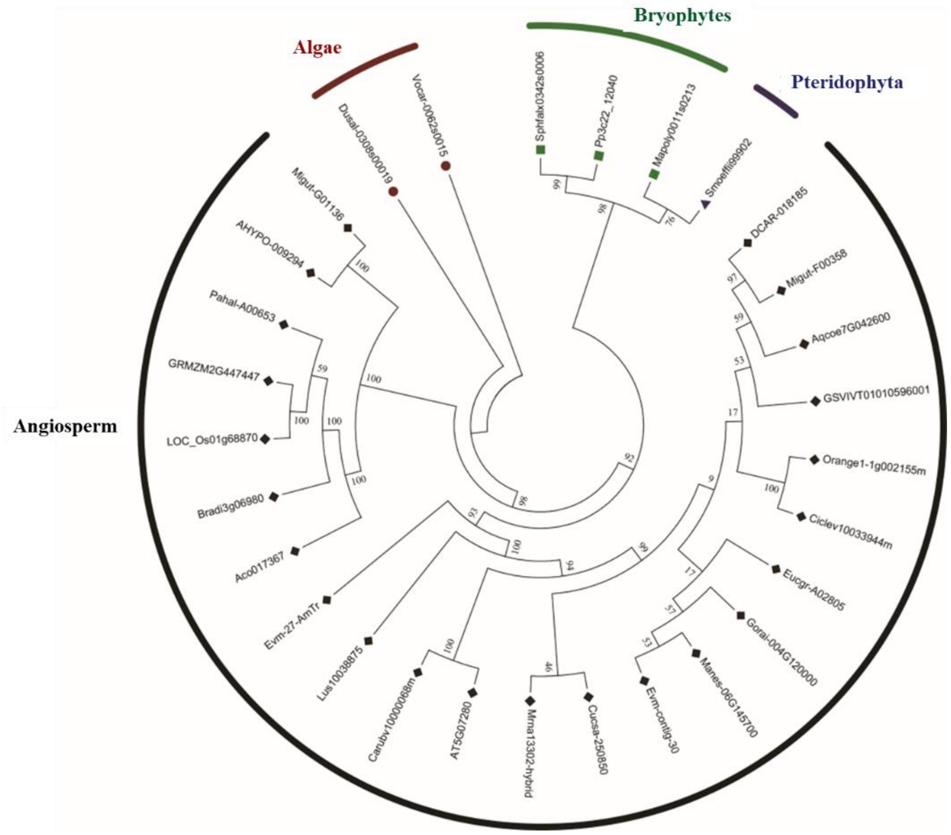

Figure S2. Bootstrapped maximum-likelihood phylogenies of the *SARK* gene family in sequenced land plants.

Table S1. Primers used for this article.

Primer name	Primer sequence (5'–3')	Description
PpSARK uF	ATGAATATCTCCAATGTAGTACAGCTTG	Cloning for *PpSARK*
PpSARK uR	CCCTCG TAGCGCTTCAATTTCA	Cloning for *PpSARK*
PpSARK dF	GTGCTTGACCTCGCTTTCAATG	Cloning for *PpSARK*
PpSARK dR	TTGTAATTCGAGG ATCGTCTTG GA	Cloning for *PpSARK*
PpSARK F1	GCCAATCGTTAGCCACAAGGAA	Genotyping
PpSARK R1	GAGTAGGGGGGCATAACTTCG	Genotyping
PpSARK F2	GTTCCCTTTAGTGAGGGTTAAT	Genotyping
PpSARK R2	TTCAGATCTGTGAAGATTAGCA	Genotyping
PpSARK F	CGCTGATGAAATTCTCCGCA	qRT-PCR
PpSARK R	CAGTGAGGTTGTTGTGCGAA	qRT-PCR
PpABI3 F	AAAGAAGCGACAATTTGCGTC	qRT-PCR
PpABI3 R	CAGATTGTCTTTGCCGCGAT	qRT-PCR
PpABI5 F	AGGCGACCATAGAAGCAA	qRT-PCR
PpABI5 R	TCCCCACCATCGGACCAG	qRT-PCR
PpPP2C F	GCCTGGCGTTTTATTTCAGCA	qRT-PCR
PpPP2C R	CCATTTCTCGTCTCCGTCCG	qRT-PCR
PpLEA F	GCGGCCCAGAAGAGCAA	qRT-PCR
PpLEA R	TTCGTCTTCTCTGGGATGTCCT	qRT-PCR
PpSAG12 F	GAGCAGTGTTTGCATCGGTT	qRT-PCR
PpSAG12 R	CCGTGTACATCCTCCGGAAC	qRT-PCR
PpAPT F	AGTATAGTCTAGAGTATGGTACCG	qRT-PCR
PpAPT R	TAGCAATTTGATGGCAGCTC	qRT-PCR

Primers located in upstream and downstream sites used for genotyping, as indicated in Figure S1, and for qRT-PCR analysis. The upstream (the first 506 bp from ATG) and downstream (the last 513

bp from TAA) fragments of *PpSARK* were amplified using genomic DNA for construct of the vector pTN182. The primers used are as follows: upstream fragment—*PpSARK* uF and *PpSARK* uR; downstream fragment—*PpSARK* dF and *PpSARK* dR (Figure S1). *PpSARK* F1 and *PpSARK* R1, and *PpSARK* F2 and *PpSARK* R2 were used for genotyping of *PpSARKg* genomic DNA samples, shown in Figure 2A. *PpSARK* F and *PpSARK* R were the primers used for qRT-PCR analysis of *PpSARKg* (Figure 2B). *PpABI3* F and *PpABI3* R, *PpABI5* F and *PpABI5* R, *PpPP2C* F and *PpPP2C* R, *PpLEA* F and *PpLEA* R, *PpSAG12* F and *PpSAG12* R, and *PpAPT* F and *PpAPT* R were the primers used for qRT-PCR analysis of *PpABI3*, *PpABI5*, *PpPP2C*, *PpLEA*, *PpSAG12*, and *PpAPT* (adenine phosphoribosyl transferase gene), respectively.
